# The Effects of Eccentric Strength Training on Flexibility and Strength in Healthy Samples and Laboratory Settings: A Systematic Review

**DOI:** 10.3389/fphys.2022.873370

**Published:** 2022-04-25

**Authors:** Sebastian Vetter, Axel Schleichardt, Hans-Peter Köhler, Maren Witt

**Affiliations:** ^1^ Department of Biomechanics in Sports, Faculty of Sports Science, Leipzig University, Leipzig, Germany; ^2^ Department of Biomechanics, Institute for Applied Training Science, Leipzig, Germany

**Keywords:** fascicle lengthening, flexibility training, athletic training, muscle adaptation, resistance training, injury prevention

## Abstract

**Background:** The risk of future injury appears to be influenced by agonist fascicle length (FL), joint range of motion (ROM) and eccentric strength. Biomechanical observations of the torque-angle-relationship further reveal a strong dependence on these factors. In practice, a longer FL improves sprinting performance and lowers injury risk. Classical stretching is a popular and evidenced-based training for enhancing ROM but does not have any effects on FL and injury risk. However, recent studies show that eccentric-only training (ECC) improves both flexibility and strength, and effectively lowers risk of injury.

**Objectives:** To review the evidence on benefits of ECC for flexibility and strength.

**Methods:** COCHRANE, PUBMED, SCOPUS, SPOLIT, and SPONET were searched for laboratory trials that compare ECC to at least one comparison group. Studies were eligible if they examined both strength and flexibility metrics in a healthy sample (<65 years) and met criteria for controlled or randomized clinical trials (CCT, RCT). 18 studies have been included and successfully rated using the PEDro scale.

**Results:** 16 of 18 studies show strong evidence of strength and flexibility enhancements for the lower limb. While improvements between ECC and concentric training (CONC) were similar for eccentric (+19 ± 10% vs. +19 ± 11%) and isometric strength (+16 ± 10% vs. +13 ± 6%), CONC showed larger improvements for concentric strength (+9 ± 6% vs. +16 ± 7%). While for ROM ECC showed improvements (+9 ± 7%), no results could be found for CONC. The overall effectiveness of ECC seems to be higher than of CONC.

**Conclusion:** There is clear evidence that ECC is an effective method for changes in muscle architecture, leading to both flexibility and strength improvements for the lower limb. Due to limited data no shoulder study could be included. Further research is needed for the upper body joints with a focus on functional and structural adaptions.

**Systematic Review Registration:** https://www.crd.york.ac.uk/prospero/display_record.php?ID=CRD42021283248, identifier CRD42021283248

## 1 Introduction

High performance sports set high demands on physical abilities due to repeated high loads and limited recovery time. To be able to compete on a high level, a professional athlete needs to train several hours a day to reach a higher performance level. This amount of chronic stress leads to degenerations, lesions and injuries which have been confirmed for lower (Le Gall et al., 2006; Aicale et al., 2018) and upper body joint structures (Luime et al., 2004; Myklebust et al., 2013). While strength training is commonly associated with performance improvements, flexibility training usually serves for recovery purposes and often is cut short in an athlete’s schedule. Therefore, it is important that athletic training aims for simultaneous and multiple effects on flexibility and strength to save time, reduce risk of injury and interruptions of training or so-called time-loss injury (Achenbach and Luig, 2020).

Irrespective of any specific joint, it has been shown that flexibility and strength are highly modifiable (O’Sullivan et al., 2012; Franchi et al., 2017; Gérard et al., 2020). Considering the fact that the muscle-tendon-unit contributes 51%, and therefore the greatest portion, to a joint’s flexibility (Johns and Wright, 1962), muscle training has great potential for ensuring both high performance and injury prevention. Especially for muscle controlled joints, like the shoulder, multi-effective intervention strategies (Fredriksen et al., 2020) are required to avoid prominent injuries like the “throwing-shoulder” (Astolfi et al., 2015) in overhead sports.

Common prevention and rehabilitation strategies are stretching, as conventional flexibility training, and concentric training (CONC). A combination of both prevention strategies requires a lot of training time but lacks effectiveness (Fredriksen et al., 2020). Examined for the lower limb, neither stretching (Konrad and Tilp, 2014; Lima et al., 2015) nor concentric training (Gérard et al., 2020) show evidence of fascicle lengthening. If reduced fascicle length (FL) is associated with increased injury risk (Timmins et al., 2016a) stretching seems to be inappropriate for injury prevention (Thacker et al., 2004). In contrast, eccentric training (ECC) for the lower limb additionally aims for flexibility-modification and leads to an increased FL (O’Sullivan et al., 2012) as well as isokinetic torque gains (Medeiros et al., 2021). ECC also has a higher impact on concentric torque than CONC does on eccentric torque (Blazevich et al., 2007). Due to its strong impact on muscular excursion range and eccentric torque, ECC might have two major effects: 1) enhanced motor performance due to an improvement of the acceleration way and torque production which might also lead to a lowered risk of injury for any joint; 2) enhanced muscular energy absorption in the decelerating limb after highly demanding concentric actions with a buffering effect on surrounding structures. The first assumption is based on research showing that ECC lowers the risk of injury by up to 70% (Timmins et al., 2016a; Ribeiro-Alvares et al., 2020) and has benefits on motor performance (Kumagai et al., 2000). The second assumption is based on higher flexibility benefits in muscles compared to various tissues (Johns and Wright, 1962; Fouré et al., 2013).

The existing systematic reviews and meta-analyses (O’Sullivan et al., 2012; Douglas et al., 2017; Medeiros et al., 2021) have not included concurrent multivariate effects on flexibility and strength and are solely focusing on the lower limb. Furthermore, most of the reviewed studies have incomparable study designs because of different definitions of eccentric training and insufficiently detailed descriptions of the eccentric training stimulus (e.g. Nelson and Bandy, 2004; Fouré et al., 2013). Also, many studies of flexibility strength training have low methodological ratings and provide confusing data (Thacker et al., 2004; Toigo and Boutellier, 2006).

Therefore, the aim of this review is to investigate whether ECC is capable of improving both strength and flexibility within highly standardized settings and therefore extend existing knowledge on the effects of ECC (O’Sullivan et al., 2012; Franchi et al., 2017). For this purpose, the authors reviewed longitudinal studies that compared ECC to a comparison group in healthy adults. All reviewed studies meet RCT or CCT criteria. Parameters such as torque, force or load for strength and range of motion (ROM) for flexibility were compared between the different interventions. Due to this approach, we intend to highlight the multidirectional effect of ECC and, thus, its significance for injury prevention and performance enhancement.

## 2 Methods

This systematic review is reported in accordance with the PRISMA-statement (Moher et al., 2009) and is registered (CRD42021283248) in PROSPERO database.

### 2.1 Eligibility Criteria

This systematic review includes peer-reviewed RCT and CCT from 1999 to 2021. According to the PICOS eligibility criteria (Methley et al., 2014) studies have to show the following factors to be eligible for analysis:

• Population: healthy male or female adults (18–65 years of age), free of injury or neuronal disease, and recreationally active or used to strength training.
• Intervention: eccentric-only training (very low to no load in the concentric phase) within a laboratory setting with a training volume of at least two sessions per week
• Control: CONC, stretching or non-intervention control group
• Outcomes: flexibility (ROM and/or FL) and strength (torque, force, or load, and/or pennation angle [PA]).
• Study design: longitudinally studies (at least 4 weeks), measuring long-term effects on RCT or CCT.

Articles that did not meet the inclusion criteria were excluded from this systematic review.

### 2.2 Search Strategy and Selection Process

**FIGURE 1:**
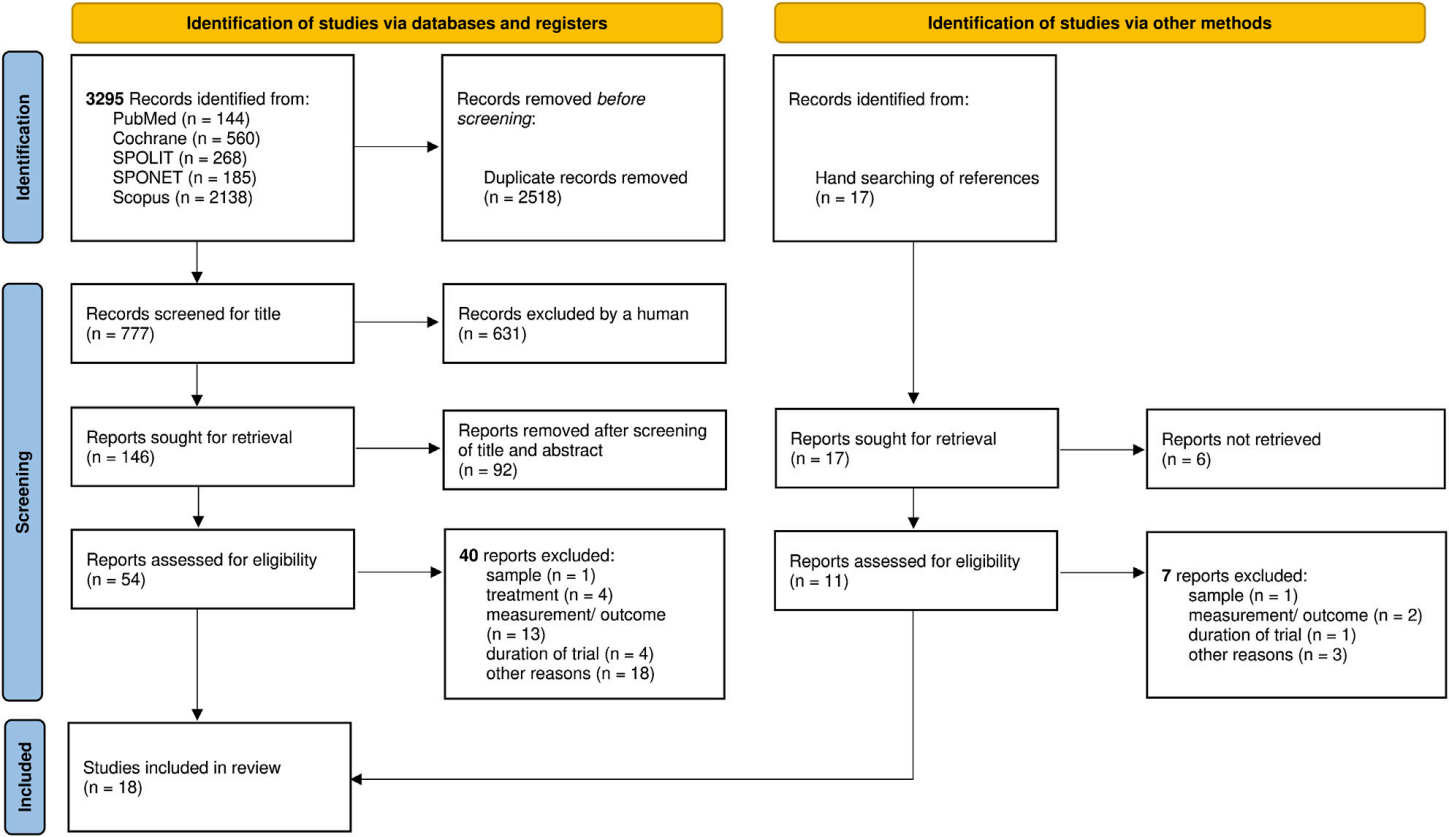
Flow diagram of selection process.

COCHRANE, PUBMED, SCOPUS, SPOLIT, and SPONET were searched by one author (SV). The search syntax included three search groups: eccentric (in abstract/title), flexib* (in abstract/title) and strength (in full text). As described in Table 1, the search groups were linked with the operator “AND”. Within each group “OR” was used to widen the search by using further synonyms. With the final syntax of step 29 (Table 1), the search was implemented in all databases. All searches were filtered for RCT, CCT, and publication date (2011–2021). Since the review of O’Sullivan et al*.* (2012) already included a systematic literature search from 1999–2010 with the same aim of research, we included its selected studies after the screening phase (described in Figure 1) and selected those studies who met the eligibility criteria of our systematic review.

**TABLE 1:** Example for the development of search syntax.

Step	Search terms (June 2020)	N in PubMed
		Filtered: RCT, CCT, 10 years
1	Eccentric^a^ (T/A)	556
2	Ecc^a^ (T/A)	165
3	Exzentrisch^a^ (T/A)	0
4	(1) OR (2) OR (3)	671
5	Flexib^a^ (T/A)	2,231
6	“Range of motion” (T/A)	1865
7	“Range of movement” (T/A)	142
8	“Joint range” (T/A)	71
9	“Joint angle” (T/A)	37
10	“Fascicle length” (T/A)	45
11	“Fascicle angle” (T/A)	4
12	Rom (T/A)	776
13	FL (T/A)	229
14	Lengthening (T/A)	129
15	Elongation (T/A)	107
16	Stretch^a^ (T/A)	1,028
17	Expan^a^ (T/A)	2,381
18	(5) OR … (17)	7,784
19	Strength	8,754
20	Training	43,646
21	Loading	3,143
22	Workout	116
23	Intervention	194,619
24	Exercise	25,005
25	Session	17,425
26	Krafttraining^a^	4
27	Übung^a^	2
28	(19) OR … (27)	201,812
29	(4) AND (18) AND (28)	122

a search for all kinds of word-endings; (T/A), search in title and abstract; (1), includes results of step 1; AND, operator AND combines all search groups; CCT, controlled clinical trial; N, number of records; OR, operator OR combines search terms of specific search step; RCT, randomized controlled trial.

Study selection included three major steps (Figure 1). After identification of studies, first and fast selection was based on title. If a title clearly showed a different topic or focus, other languages than English or German, the study was excluded. The second step was based on title and abstract. Studies were excluded if they conducted experiments on animals, focused on post-surgery training, training of injured athletes or did not focus on flexibility and strength. Full-text screening included a check for defined eligibility criteria. Therefore, studies were excluded if they showed insufficient ECC or testing protocols for flexibility and strength metrics, no laboratory settings, no comparison group, and if the full text could not be obtained from database or authors.

### 2.3 Data Extraction

Two authors (SV, HPK) independently extracted and cross-checked the following data:

a) Studies’ characteristics: authors and year, specifics of study, participants, activity level and health status, outcome variables,
b) Training protocol: training groups, exercises, number of training weeks and sessions, within training the number of sets and repetitions, intensity of training, range of motion and duration of each movement,
c) Methodological quality: description of exclusion criteria, randomization, concealment, baseline values, blinded subjects, blinded therapist, blinded assessor, follow-up/post-test, intention to treat, between group analysis, point measures and variability,
d) Results: FL, ROM, PA, eccentric, concentric and isometric strength.

### 2.4 Assessment of Methodological Quality

As a reliable (Maher et al., 2003) and valid tool (de Morton, 2009) for rating methodological quality of studies, the PEDro scale was used independently by two authors (SV, HPK). The third author (AS) cross-checked the results and all three authors reached consensus. The classification of study quality can reach from “poor” (<4/11) over fair (4–5/11) to high (>6/11). A publication bias exists because articles were only searched in online databases. Performance criteria for flexibility and strength measurements were appraised and lead to a pre-selection of studies due to strict eligibility criteria.

### 2.5 Data Synthesis

All joints and muscle groups were analyzed together to show consistent evidence of ECC on any joint’s flexibility and strength. Due to the amount of data, simple pooled analyses were conducted to summarize results for a quick overview. Complex pooled analyses were not part of this systematic review.

## 3 Results

### 3.1 Study Selection

As outlined in Figure 1, a total of 3,295 studies were identified via databases and registers as potentially relevant papers. After manually removing duplicates 777 records remained. The manual screening for title excluded 631 records. 146 records were screened for title and abstract. 54 reports were eligible for full-text analyses.

Additionally, the hand-search of references revealed 17 records, of which 6 were included in the systematic search of O’Sullivan et al*.* (2012) and of which 11 originated from different articles. The screening for title and abstract revealed 11 studies eligible for full-text analyses. 54 + 11 records met criteria for full-text analysis of which 47 were removed including 3 articles of O’Sullivan et al*.* (2012). In the end a total of 18 studies met all inclusion criteria (Blazevich et al., 2007; Duclay et al., 2009; Potier et al., 2009; Guilhem et al., 2013; Franchi et al., 2014; Sharifnezhad et al., 2014; Coratella et al., 2015; Timmins et al., 2016b; Bourne et al., 2017; Seymore et al., 2017; Abdel-Aziem et al., 2018; Kay et al., 2018; Ribeiro-Alvares et al., 2018; Duhig et al., 2019; Delvaux et al., 2020; Marušič et al., 2020; Benford et al., 2021; Uysal et al., 2021) and were included in this systematic review.

### 3.2 Description of Included Studies

Presented in Table 2, the number of participants ranged from *n* = 12 (Franchi et al., 2014) to *n* = 60 subjects (Abdel-Aziem et al., 2018). Samples’ mean age ranged from 19 (Kay et al., 2018) to 28 years (Potier et al., 2009; Seymore et al., 2017). 11 of the 18 studies included males only (Duclay et al., 2009; Guilhem et al., 2013; Franchi et al., 2014; Coratella et al., 2015; Timmins et al., 2016b; Bourne et al., 2017; Abdel-Aziem et al., 2018; Duhig et al., 2019; Delvaux et al., 2020; Benford et al., 2021; Uysal et al., 2021). 15 studies examined FL changes by ultrasound (Blazevich et al., 2007; Duclay et al., 2009; Potier et al., 2009; Guilhem et al., 2013; Franchi et al., 2014; Sharifnezhad et al., 2014; Coratella et al., 2015; Timmins et al., 2016b; Bourne et al., 2017; Seymore et al., 2017; Kay et al., 2018; Ribeiro-Alvares et al., 2018; Duhig et al., 2019; Marušič et al., 2020; Benford et al., 2021). 6 studies measured ROM either with isokinetic dynamometer (Potier et al., 2009; Kay et al., 2018), sit-and-reach-test (Ribeiro-Alvares et al., 2018; Uysal et al., 2021), a 3D-video recording (Delvaux et al., 2020), or a goniometer (Abdel-Aziem et al., 2018). 3 studies measured FL and ROM concurrently (Potier et al., 2009; Kay et al., 2018; Ribeiro-Alvares et al., 2018).

**TABLE 2:** Studies’ characteristics.

Study	Specifics of study	Participants (mean age)	Activity level/Health status	Outcome flexibility	Outcome strength
Abdel-Aziem et al. (2018)	Trained vs. untrained	m (*n* = 60); 21 year	Active; > 1 year without injury/complications	ROM	MVC
Benford et al. (2021)	CONC vs. ECC; region specific changes	m (*n* = 16); 23 year	Active (>1 h/day; at least 3 days/week); no injuries	FL	MVC, iMVC, PA
Blazevich et al. (2007)	CONC vs. ECC; follow-up	w/m (*n* = 33); 23 year	Active; no history of lower limb injury; no co-existing medical condition	FL	MVC, PA
Bourne et al. (2017)	NHE vs. hip extensions; allocation by FL	m (*n* = 30); 22 year	Active; no injury/strain for structures next to core, hips or knee	FL	load (kg)
Coratella et al. (2015)	IK vs. IL	m (*n* = 49); 20year	>6 months no strength training; no knee injury/complication	FL	MVC, iMVC, load (kg) PA
Delvaux et al. (2020)	Four field exercises	m (*n* = 27); 23 year	Active; Sports with running actions; No injury of lower limb or history of knee surgery	ROM	MVC
Duclay et al. (2009)	Examining active and passive stiffness	m (*n* = 18); 23 year	Active; students without neurological injury/disease	FL	iMVC, PA
Duhig et al. (2019)	CONC vs. ECC; allocation by FL	m (*n* = 30); 23 year	Active; no history of hamstrings/knee injury	FL	iMVC, load, PA
Franchi et al. (2014)	CONC vs. ECC; allocation by strength	m (*n* = 12); 25 year	Untrained; health status not reported	FL	iMVC, PA
Guilhem et al. (2013)	IK vs. IL	m (*n* = 31); 20 year	Untrained; healthy; no history of knee injury	FL	MVC, iMVC,PA
Kay et al. (2018)	Examining tendon stiffness and energy storage; allocation by sex	w/m (*n* = 26); 28 year	Active; no history of lower limb injury	ROM, FL	MVC, iMVC, PA
Marušič et al. (2020)	NHE-modified for more stretching; sample has to be able to control at least 50% of ROM of NHE	w/m (*n* = 34); 24 year	Active (>3 h of physical activity/wk); >12 months free from neural, muscular, skeletal, or connective tissue injuries	FL	MVC, iMVC, PA
Potier et al. (2009)	Age 20–50 years	w/m (*n* = 22); 28 year	No musculoskeletal injury; no co-existing medical conditions	ROM, FL	load (kg), PA
Ribeiro-Alvares et al. (2018)	NHE	w/m (*n* = 20); 25 year	Active; >1 year no lower limb injury; no history of hamstrings strain	ROM, FL	MVC, iMVC, PA
Seymore et al. (2017)	NHE	w/m (*n* = 20); 19 year	Active; no history of hamstrings injuries; No BMI >30 kg/m^2^	FL	MVC
Sharifnezhad et al. (2014)	Studying dose-response-relationship	w/m (*n* = 31); 27 year	—	FL	iMVC
Timmins et al. (2016b)	CONC vs. ECC; follow-up; allocation by FL	m (*n* = 28); 22 year	Active; > 1 year no injury of lower limb	FL	MVC, PA
Uysal et al. (2021)	NHE vs. CONC vs. NMES; relation between visco-elastic properties and flexibility/strength changes	m (*n* = 40); 22 year	Active (<2x/week); no musculoskeletal or neurological problems, chronic pain, restrictions to execute exercise	ROM	iMVC

—, not reported; <, less than; >, more than, CONC, concentric training group; d, days; ECC, eccentric training group; m, men; n, sample size; FL, fascicle length; iMVC, isometric MVC; IK, isokinetic training; IL, isoload training; mo, months; MVC, maximum voluntary contraction; NHE, nordic hamstrings exercise; NMES, electronical stimulation of muscle; PA, pennation angle; ROM, range of motion; w, women; wk, weeks; vs., versus; y, age in years.

All studies examined strength changes from pre to post intervention. 11 studies tested for isometric strength (Duclay et al., 2009; Guilhem et al., 2013; Franchi et al., 2014; Sharifnezhad et al., 2014; Coratella et al., 2015; Kay et al., 2018; Ribeiro-Alvares et al., 2018; Duhig et al., 2019; Marušič et al., 2020; Benford et al., 2021; Uysal et al., 2021). 10 studies tested isokinetic strength (Blazevich et al., 2007; Guilhem et al., 2013; Coratella et al., 2015; Timmins et al., 2016b; Seymore et al., 2017; Abdel-Aziem et al., 2018; Kay et al., 2018; Ribeiro-Alvares et al., 2018; Delvaux et al., 2020; Marušič et al., 2020). 2 studies used field exercises for training and testing (Bourne et al., 2017; Duhig et al., 2019). 1 study (Potier et al., 2009) tested for a 1RM with a hamstrings curls machine. Injured or previously injured samples were not part of any selected study. Eligibility criteria were similar between studies.

### 3.3 Characteristics of Eccentric Training

Training protocols were very heterogeneous between studies. 13 studies compared effects of a training group versus a non-training control group (Blazevich et al., 2007; Duclay et al., 2009; Potier et al., 2009; Guilhem et al., 2013; Sharifnezhad et al., 2014; Coratella et al., 2015; Bourne et al., 2017; Seymore et al., 2017; Abdel-Aziem et al., 2018; Kay et al., 2018; Ribeiro-Alvares et al., 2018; Delvaux et al., 2020; Marušič et al., 2020). Timmins et al. (2016b) showed a non-training control limb. 11 studies showed more than one training group, thereof 6 studies had a CONC comparison-group (Blazevich et al., 2007; Franchi et al., 2014; Timmins et al., 2016b; Duhig et al., 2019; Benford et al., 2021; Uysal et al., 2021), and 4 studies compared different ECC protocols (Guilhem et al., 2013; Sharifnezhad et al., 2014; Coratella et al., 2015; Bourne et al., 2017; Abdel-Aziem et al., 2018). 7 of 18 studies trained participants with an isokinetic dynamometer (Blazevich et al., 2007; Guilhem et al., 2013; Sharifnezhad et al., 2014; Coratella et al., 2015; Timmins et al., 2016b; Kay et al., 2018; Benford et al., 2021), 8 with other machines (Duclay et al., 2009; Potier et al., 2009; Franchi et al., 2014; Coratella et al., 2015; Bourne et al., 2017; Abdel-Aziem et al., 2018; Marušič et al., 2020; Uysal et al., 2021), and 7 studies used different eccentric field exercises (Bourne et al., 2017; Seymore et al., 2017; Ribeiro-Alvares et al., 2018; Duhig et al., 2019; Delvaux et al., 2020; Marušič et al., 2020; Uysal et al., 2021).

The average duration of intervention was 7.1 ± 2 weeks, ranging from 4 (Ribeiro-Alvares et al., 2018) to 10 weeks (Blazevich et al., 2007; Franchi et al., 2014; Sharifnezhad et al., 2014; Bourne et al., 2017). An average of 2.7 ± 0.8 sessions per week was documented, ranging from 1 (Seymore et al., 2017; Duhig et al., 2019; Uysal et al., 2021) to 5 sessions (Abdel-Aziem et al., 2018). Only 3 studies showed interventions lasting less than 6 weeks (Ribeiro-Alvares et al., 2018; Duhig et al., 2019; Benford et al., 2021). The average number of sets was 4.5 ± 1.2 sets with a minimum of 2 sets (Bourne et al., 2017; Seymore et al., 2017; Duhig et al., 2019; Delvaux et al., 2020; Marušič et al., 2020; Uysal et al., 2021) and a maximum of 6 sets (Blazevich et al., 2007; Duclay et al., 2009; Timmins et al., 2016b; Bourne et al., 2017; Abdel-Aziem et al., 2018). Repetitions per set show an average of 9.1 ± 2.8 repetitions, ranging from 5 (Seymore et al., 2017; Abdel-Aziem et al., 2018; Marušič et al., 2020; Uysal et al., 2021) to 16 repetitions (Sharifnezhad et al., 2014). Duration of a repetition ranged from less than 1 s (Sharifnezhad et al., 2014) to 5 s (Potier et al., 2009; Marušič et al., 2020). Training intensity ranged from 40% (Abdel-Aziem et al., 2018) to at least 100% of the concentric 1RM (Duclay et al., 2009; Potier et al., 2009; Guilhem et al., 2013; Sharifnezhad et al., 2014; Coratella et al., 2015; Timmins et al., 2016b; Delvaux et al., 2020; Benford et al., 2021; Uysal et al., 2021). 4 studies did not report training intensities (Seymore et al., 2017; Ribeiro-Alvares et al., 2018; Duhig et al., 2019; Marušič et al., 2020). ROM during training varied between 40° (Sharifnezhad et al., 2014) and 85° or full ROM (Blazevich et al., 2007; Potier et al., 2009; Franchi et al., 2014; Coratella et al., 2015; Timmins et al., 2016b; Bourne et al., 2017; Kay et al., 2018; Duhig et al., 2019). Table 3 presents further details on training characteristics.

**TABLE 3:** Eccentric training protocols.

Study	Exercises	Weeks x sessions/Pause	Sets x reps/Pause	Intensity (%1RM)	ROM & duration
Abdel-Aziem et al. (2018)	Pulley system; horizontal; p.f. hips (90°), p.m. knee	6 × 5/-	6 × 5/-	40%	—
Benford et al. (2021)	IsoDyn:	5 × 2/>48 h	4 × 8/60 s	All-out	—; 30°/s
Blazevich et al. (2007)	IsoDyn; seated; p.f. hips; p.m. knee	10 × 3/>24 h	4>6 × 6/60 s	90%ecc/conc	85°; 3 s
Bourne et al. (2017)	ECC NHE: p.f. hips, p.m. knee; ECC HE: Hip extension machine (45°): p.f. knee, p.m. hips	10 × 2/>48 h	2>6 × 6>10/120 s	60–80%	90°; —
Coratella et al. (2015)	ECC IK: isoDyn; ECC IL: knee extension machine	6x2/>48 h	5 × 8/120 s	120%conc	85°; —
Delvaux et al. (2020)	NHE; three other exercises: p.f. knee, p.m. hips	6x2-3/>48 h	2>3 × 6>10/120 s	All-out	—
Duclay et al. (2009)	Calf machine or leg press, full support for weight return; p.f. knee, p.m. foot	7x2-3/-	6 × 6/180 s	120%conc	50–60°; ∼3 s
Duhig et al. (2019)	NHE	5 × 2<1/>48 h	2>5 × 6/-	-	80–90°; -
Franchi et al. (2014)	Leg press, full support for weight return; p.f. knee, p.m. foot	10 × 3/-	4x8-10/60 s	80%	—; 2–3 s
Guilhem et al. (2013)	IsoDyn; horizontal; p.f. hips, p.m. knee	9 × 2/—	3>5 × 8/—Controlled by total work	100%conc/all-out	60°; —
Kay et al. (2018)	IsoDyn; seated; p.f. hips, p.m. knee	6 × 2/>48 h	5 × 12/60 s	>80%	90°; 3 s
Marušič et al. (2020)	NHE-modified: p.f. hips 75°, p.m. knee; Askling’s-glider: p.f. knee, p.m. hips	6 × 2/—	2>3 × 5>8/—	—	>50%ROM; 3–5s
Potier et al. (2009)	Hamstrings-curls; p.f. hips, p.m. knee	8 × 3/—	3 × 8/—	100%ecc	Maximum; 5 s
Ribeiro-Alvares et al. (2018)	NHE	4 × 2/—	3x6-10/60 s	—	—, @4 s
Seymore et al. (2017)	NHE	6 × 1>3/—	2>3 × 5>12/—	—	—
Sharifnezhad et al. (2014)	1–4) ECC low load, high load, low ROM, fast: isoDyn; p.f. hip, p.m. knee; subject 1: protocol 1 & 2, subject 2: 3 & 4	10 × 3/—	1) 5 × 10 2) 5 × 6 3) 5 × 12 4) 5 × 16 Controlled by total work	1) 65%ecc 2–4) 100%	1–4) 75°; ∼1 s 1) 40°; <0,5 s
Timmins et al. (2016b)	IsoDyn; seated; p.f. hips (85°), p.m. knee	6 × 2>3/>48 h	4>6 × 6>8/30 s	All-out	90°; <1.5 s
Uysal et al. (2021)	ECC NHE; CONC: hamstrings leg curl machine, lying, p.f. hips, p.m. knee	8 × 1>3/>24 h	2>3 × 5>12/120 s	All-out until full ROM reached	—

—, not reported; °, degrees of angle; >, more than; CONC, concentric training; conc, concentric strength; CG, control group; ECC, eccentric training; ecc, eccentric strength; h, hours; HE, hip extensions exercise; IK, isokinetic mode; IL, isoload mode; isoDyn, isokinetic dynamometry; NHE, Nordic hamstrings exercise; p.f., punctum fixum; p.m., punctum mobile; RM, repetition maximum; ROM, range of motion; s, seconds; x, times

### 3.4 Methodological Quality

Quality results are presented in Table 4. Based on the PEDro scale, every study received “high quality” rating ranging from 6 to 9 points. All studies were conducted under a concealed condition. Except Sharifnezhad et al. (2014), all studies reported clearly their exclusion criteria for the investigated groups. Apart from Abdel-Aziem et al. (2018) who divided the sample in advance according to the factor “training level”, the rest of the included studies showed clear characteristics of a randomized controlled trial (RCT). 5 studies reported specific allocation methods to equalize groups for strength (Blazevich et al., 2007; Franchi et al., 2014) or FL (Timmins et al., 2016b; Bourne et al., 2017; Duhig et al., 2019). 3 studies reported baseline differences between groups (Potier et al., 2009; Franchi et al., 2014; Duhig et al., 2019). This might affect adaptability of each group and could also explain different outcomes (see discussion). Blinding did not take part in any trial with exception of blinding the assessor in 3 studies (Guilhem et al., 2013; Bourne et al., 2017; Delvaux et al., 2020). All trials reported follow-up tests with at least 85% of participants as well as “between group analysis” (BGA) and “point measurements and variability” (PMV). 2 studies did not fulfill the intention-to-treat criteria (Sharifnezhad et al., 2014; Kay et al., 2018).

**TABLE 4:** Rating of methodological quality.

Study	Exclusion criteria	Random	Conceal	Baseline	Blind subject	Blind therapist	Blind assessor	Follow-up	ITTA	BGA	*PMV*	Score
Abdel-Aziem et al. (2018)	1	0	1	1	0	0	0	1	1	1	1	**7**
Benford et al. (2021)	1	1	1	1	0	0	0	1	1	1	1	**8**
Blazevich et al. (2007)	1	1	1	1	0	0	0	1	1	1	1	**8**
Bourne et al. (2017)	1	1	1	1	0	0	1	1	1	1	1	**9**
Coratella et al. (2015)	1	1	1	1	0	0	0	1	1	1	1	**8**
Delvaux et al. (2020)	1	1	1	1	0	0	1	1	1	1	1	**9**
Duclay et al. (2009)	1	1	1	1	0	0	0	1	1	1	1	**8**
Duhig et al. (2019)	1	1	1	0	0	0	0	1	1	1	1	**7**
Franchi et al. (2014)	1	1	1	0	0	0	0	1	1	1	1	**7**
Guilhem et al. (2013)	1	1	1	1	0	0	1	1	1	1	1	**9**
Kay et al. (2018)	1	1	1	1	0	0	0	1	0	1	1	**7**
Marušič et al. (2020)	1	1	1	1	0	0	0	1	1	1	1	**8**
Potier et al. (2009)	1	1	1	0	0	0	0	1	1	1	1	**7**
Ribeiro-Alvares et al. (2018)	1	1	1	1	0	0	0	1	1	1	1	**8**
Seymore et al. (2017)	1	1	1	1	0	0	0	1	1	1	1	**8**
Sharifnezhad et al. (2014)	0	1	1	1	0	0	0	1	0	1	1	**6**
Timmins et al. (2016b)	1	1	1	1	0	0	0	1	1	1	1	**8**
Uysal et al. (2021)	1	1	1	1	0	0	0	1	1	1	1	**8**

0, does not meet criteria; 1, meets criteria; BGA, between-group-analysis; ITTA, intention to-treat analysis; PMV, point measure and variability.

Bold values: total score for each study.

### 3.5 Description of Results

Table 5 shows the results of the 18 included studies. Since different muscle groups are involved, percentage changes in each parameter were extracted from all studies. If not provided by the authors, pre-post values were used for calculation of percentage change (underlined letters, Table 5). Based on these values, simple pooled analyses were conducted for ECC, CONC and control group (CG).

**TABLE 5:** Study results.

Study	Groups	∆ Eccentric strength	∆ Concentric strength	∆ Isometric strength	∆ ROM	∆ PA	∆ FL
Abdel-Aziem et al. (2018)	1) ECC trained	**7.6%** IK 60°/s	0.6%	—	**−11.4%**	—	—
	2) ECC untrained	**15%**	1,5%	—	**−19.6%**	—	—
	3) CG	0.6%	1.3%	—	**−** 2.8%	—	—
Benford et al. (2021)	1) ECC	**12.61%**	**12.31%**	**11.42%** @ 70°	—	4.09%*	7.23%*
	2) CONC	**13.41%**	**11.09%**	**10.71%**	—	**1.36%** distal-end	0%
Blazevich et al. (2007)	1) ECC	**38.9 ± 14.2%** : IK 30°/s	**16.4 ± 5.1%**	—	—	**21.4 ± 6.9%** VL	**3.1 ± 1.6%**
	2) CONC	**35.9 ± 12.7%**	24.1 ± 4.2%*	—	—	13.3 ± 3.0%	**6.3 ± 3.0%**
	3) CG	3.0 ± 3.2%	0.5 ± 1.8%	—	—	0.4 ± 3.6% VL	−0.3 ± 0.9%
Bourne et al. (2017)	1) ECC NHE	***97.38 N/26 kg*** NHE/HE	—	—	—	—	***2.22* *cm***
	2) ECC HE	***110.47 N/41*** * * ***kg***	—	—	—	—	***1.33 cm***
	3) CG	8.91 N/3.50 kg	—	—	—	—	
Coratella et al. (2015)	1) ECC IK	32.4%* IK 60°/s	**7.7%**	**14.4%**	—	11.6%	**15.1%**
	2) ECC IL	**12.9%**	**5.2%**	**5.9%**	—	4.6%	**14%**
	3) CG	−3.8%	−4.7%	−2.2%	—	4.9%	1.3%
Delvaux et al. (2020)	1) ECC	**7.1%** IK 30°/s	7.2%: IK 60°/s	—	**12.7%**	—	—
	2) CG	−3.3%	−2%	—	N.s	—	—
Duclay et al. (2009)	1) ECC	—	—	**13.3%**	—	**7.6%**	**6.8%**
	2) CG	—	—	n.s	—	—	n.s
Duhig et al. (2019)	1) ECC	**24%** NHE	—	—	—	−5%	**13%**
	2) CONC	**13%**	—	—	—	12%*	−**6%**
Franchi et al. (2014)	1) ECC	—	—	**11 ± 8%**	—	5 ± 1%	12 ± 2%*
	2) CONC	—	—	**9 ± 6%**	—	30 ± 0.5%*	5 ± 1%
Guilhem et al. (2013)	1) ECC IL	+15 ± 4% IK 180°/s	18 ± 3%	**16 ± 3%**	—	11 ± 6%*	−3%
	2) ECC IK	n.s	8 ± 3%	**14 ± 3%**	—	n.s	3.4%
	3) CG	n.s	n.s	n.s	—	n.s	n.s
Kay et al. (2018)	1) ECC	**29.5 ± 15.8%** IK 30°/s	—	**17.4 ± 7.9%**	**3.5%**	**9.0 ± 2.8%**	−0.7 ± 0.9%
	2) CG	5.0 ± 2.8%	—	−6.3 ± 4.8%	−0.5%	0.8%	−0.9 ± 2.2%
Marušič et al. (2020)	1) ECC	**16.76%** IK 60°/s	**18.05%**	**16.67%**	—	**11.43%**	**7.49%**
	2) CG	0%	0.55%	−1.99%	—	0.77%	−0.5%
Potier et al. (2009)	1) ECC	**34.2%** 1RM curling	—	—	**5%**	−2.9%	**33.5%**
	2) CG	3.5%	—	—	**−** 1.2%	6.5%	16.6%
Ribeiro-Alvares et al. (2018)	1) ECC	**14.4%** IK 60°/s	12.3%	**9.6%**	0.4%	**−16,86%**	**21.77%**
	2) CG	1.2%	3.1%	**−** 2%	**−** 4.1%	**−** 1%	2%
Seymore et al. (2017)	1) ECC	11.6% IK 60°/s	—	—	—	9.6%	1.2%
	2) CG	**−** 4.6%	—	—	—	(12.4°)	0.1%
Sharifnezhad et al. (2014)	1) ECC low load	—	—	significant changes between ***30–70°***	—	—	n.s
	2) ECC high load	—	—	significant changes between ***25–65°***	—	—	n.s
	3) ECC low ROM	—	—	significant changes between ***30–60°***	—	—	n.s
	4) ECC fast	—	—	significant changes at ***45°***	—	—	14%*
	5) CG	—	—	n.s	—	—	n.s
Timmins et al. (2016b)	1) ECC	**16.6%** IK 60°/s	**16.5%**	—	—	**−7.5%**	**16.4%**
	2) CONC	**14.4%**	**13.1%**	—	—	**20.1%**	**−11.8%**
	3) CG (second limb)	—	—	—	—	—	—
Uysal et al. (2021)	1) ECC	—	—	up to 44%*	***3.64 cm*** sit and reach	—	—
	2) CONC	—	—	**20.4%**	***2.58 cm*** sit and reach	—	—
	3) NMES	—	—	**11.9%**	*0.21 cm* sit and reach	—	—
Total	1) ECC	+19 ± 10%	+9 ± 6%	+16 ± 10%	+9 ± 7%	+5 ± 7%	+10 ± 9%
	2) CONC	+19 ± 11%	+16 ± 7%	+13 ± 6%	—	+15 ± 11%	−1 ± 8%
	3) CG	−1 ± 3%	−1 ± 3%	−3 ± 2%	−2 ± 2%	2 ± 3%	+3 ± 6%

Bold letters, significant change from pre-to-post or vs. CG; underlined letters, calculated based on pre-post-values; values within brackets, possibly wrong reported; -, not reported; °, degrees of angle; ±, standard deviation; *, significant improved vs. other training-group; CG, control group; CONC, concentric training; ECC, eccentric training; FL, fascicle length; IK, isokinetic mode; n.v., no pre-post values reported; HE, hyperextension exercise; IL, isoload mode; Nm, Newton meter; NHE, Nordic hamstrings exercise; NMES, neuromuscular electro stimulation; n.s., not significant without pre-post-values reported; italic letters, no pre-post values reported; PA, pennation angle; RM, repetition maximum; ROM, range of motion; TAR, measurement of torque angle relationship each 5°; VL, m. vastus lateralis; vs.; versus.

Among the 18 studies of this systematic review, a total of 25 ECC subgroups were identified and considered for the description of results. All included studies reported at least one result for functional strength changes (either eccentric, concentric or isometric) and flexibility changes (either range of motion, sit-and-reach or fascicular lengthening). Since a change in PA is understood as a change in strength by cross-sectional hypertrophy (Franchi et al., 2017), this parameter is also listed in Table 5 as a morphological surrogate for strength.

### 3.6 Functional and Morphological Strength Changes

#### 3.6.1 Eccentric Strength

18 ECC subgroups were tested for eccentric strength, 3 groups without significant changes (Guilhem et al., 2013; Seymore et al., 2017). Results ranged from +7.1% (Delvaux et al., 2020) to +38.9% (Blazevich et al., 2007) enhancement in eccentric strength.

Simple pooled analysis of 15 ECC subgroups (Blazevich et al., 2007; Potier et al., 2009; Guilhem et al., 2013; Coratella et al., 2015; Timmins et al., 2016b; Seymore et al., 2017; Abdel-Aziem et al., 2018; Kay et al., 2018; Duhig et al., 2019; Delvaux et al., 2020; Marušič et al., 2020; Ribeiro-Alvares et al., 2020; Benford et al., 2021) showed an average improvement of +19 ± 10% of eccentric strength. 4 subgroups with CONC revealed +19 ± 11% improvement (Blazevich et al., 2007; Potier et al., 2009; Guilhem et al., 2013; Coratella et al., 2015; Seymore et al., 2017; Abdel-Aziem et al., 2018; Kay et al., 2018; Ribeiro-Alvares et al., 2018; Delvaux et al., 2020; Marušič et al., 2020). 10 inactive CGs showed no significant change (−1 ± 3%) in eccentric strength (Blazevich et al., 2007; Potier et al., 2009; Guilhem et al., 2013; Coratella et al., 2015; Seymore et al., 2017; Abdel-Aziem et al., 2018; Kay et al., 2018; Ribeiro-Alvares et al., 2018; Delvaux et al., 2020; Marušič et al., 2020).

#### 3.6.2 Concentric Strength

Concentric strength values were reported for 12 ECC subgroups, 6 of which showed no significant changes (Guilhem et al., 2013; Abdel-Aziem et al., 2018; Ribeiro-Alvares et al., 2018; Delvaux et al., 2020). Results ranged from a non-significant gain of +0.6% (Guilhem et al., 2013; Abdel-Aziem et al., 2018) to +18% (Marušič et al., 2020) enhancement in concentric strength.

11 ECC subgroups were used for simple pooled analysis (Blazevich et al., 2007; Guilhem et al., 2013; Coratella et al., 2015; Timmins et al., 2016b; Abdel-Aziem et al., 2018; Ribeiro-Alvares et al., 2018; Delvaux et al., 2020; Marušič et al., 2020; Benford et al., 2021). After ECC, concentric strength improved by +9 ± 6%. 3 CONC groups showed an mean improvement of +16 ± 7% (Blazevich et al., 2007; Timmins et al., 2016b; Benford et al., 2021). CGs reported no change (−1 ± 3%) in concentric strength (Blazevich et al., 2007; Coratella et al., 2015; Abdel-Aziem et al., 2018; Ribeiro-Alvares et al., 2018; Delvaux et al., 2020; Marušič et al., 2020).

#### 3.6.3 Isometric Strength

All of 15 ECC groups revealed isometric strength gains. Results varied between +5.9% (Coratella et al., 2015) and a significant gain of +44% (Uysal et al., 2021) after ECC. In contrast, the maximum improvement in isometric strength after CONC was +20.4% (Uysal et al., 2021).

For the simple pooled analysis, 11 ECC subgroups were included (Blazevich et al., 2007; Guilhem et al., 2013; Coratella et al., 2015; Timmins et al., 2016b; Abdel-Aziem et al., 2018; Ribeiro-Alvares et al., 2018; Delvaux et al., 2020; Marušič et al., 2020; Benford et al., 2021). ECC improved by +16 ± 10%. 3 CONC subgroups showed mean isometric strength changes of +13 ± 6% (Franchi et al., 2014; Benford et al., 2021; Uysal et al., 2021). No significant changes (−3 ± 2%) were seen in CGs (Coratella et al., 2015; Ribeiro-Alvares et al., 2018; Marušič et al., 2020).

#### 3.6.4 Pennation Angle

PA values were reported for 15 ECC subgroups, 7 of which showed no significant changes (Potier et al., 2009; Guilhem et al., 2013; Franchi et al., 2014; Coratella et al., 2015; Seymore et al., 2017; Duhig et al., 2019). Results ranged from −16.9% (Ribeiro-Alvares et al., 2018) to +21.4% (Blazevich et al., 2007).

14 ECC subgroups were used for simple pooled analysis (Blazevich et al., 2007; Duclay et al., 2009; Potier et al., 2009; Guilhem et al., 2013; Franchi et al., 2014; Coratella et al., 2015; Timmins et al., 2016b; Seymore et al., 2017; Kay et al., 2018; Ribeiro-Alvares et al., 2018; Duhig et al., 2019; Marušič et al., 2020; Benford et al., 2021). After ECC the average angular change in PA was +5 ± 7%. In comparison, 5 CONC subgroups showed an average PA change of +15 ± 11% (Blazevich et al., 2007; Franchi et al., 2014; Timmins et al., 2016b; Duhig et al., 2019; Benford et al., 2021). 6 CGs showed no significant values with an average PA change of 2 ± 3% (Blazevich et al., 2007; Potier et al., 2009; Coratella et al., 2015; Kay et al., 2018; Ribeiro-Alvares et al., 2018; Marušič et al., 2020).

### 3.7 Functional and Morphological Changes of Flexibility

#### 3.7.1 Passive Range of Motion

7 subgroups were tested for ROM, 1 of which showed no significant changes (Ribeiro-Alvares et al., 2018). Results ranged from −19.6% (Abdel-Aziem et al., 2018) up to +12.7% improvement (Delvaux et al., 2020).

Simple pooled analysis of 6 ECC subgroups showed an average ROM improvement of +9 ± 7% (Potier et al., 2009; Abdel-Aziem et al., 2018; Kay et al., 2018; Ribeiro-Alvares et al., 2018; Delvaux et al., 2020). 4 CGs revealed a mean decrease of −2 ± 2% (Potier et al., 2009; Abdel-Aziem et al., 2018; Kay et al., 2018; Ribeiro-Alvares et al., 2018). None of the studies including CONC interventions did report ROM values.

#### 3.7.2 Muscle Fascicle Length

FL values were reported for 21 subgroups, 7 of which showed no significant changes (Guilhem et al., 2013; Sharifnezhad et al., 2014; Seymore et al., 2017; Kay et al., 2018). Results ranged from a non-significant result of −3% (Guilhem et al., 2013) to a significant improvement of FL by +33.5% (Potier et al., 2009).

Simple pooled analysis revealed an average gain of FL by +10 ± 9% for 16 ECC subgroups (Blazevich et al., 2007; Duclay et al., 2009; Potier et al., 2009; Guilhem et al., 2013; Franchi et al., 2014; Coratella et al., 2015; Timmins et al., 2016b; Seymore et al., 2017; Kay et al., 2018; Ribeiro-Alvares et al., 2018; Duhig et al., 2019; Marušič et al., 2020; Benford et al., 2021). 5 CONC showed an average change of −1 ± 8% (Blazevich et al., 2007; Franchi et al., 2014; Timmins et al., 2016b; Duhig et al., 2019; Benford et al., 2021). 7 CGs reached an average FL change of +3 ± 6%, all of which were non-significant (Blazevich et al., 2007; Potier et al., 2009; Coratella et al., 2015; Seymore et al., 2017; Kay et al., 2018; Ribeiro-Alvares et al., 2018; Marušič et al., 2020). Only Benford et al*.* (2021) reported differences of FL change between ECC and CONC subgroups and found a significant higher gain in FL after ECC (+7%) than after CONC (0%).

## 4 Discussion

### 4.1 Main Findings

17 of 18 studies showed significant improvements in at least one strength parameter and 16 of 18 studies in at least one flexibility parameter after ECC. This can be seen as a very consistent result which could also be concluded from comparable review articles (Roig et al., 2009; O’Sullivan et al., 2012; Douglas et al., 2017; Franchi et al., 2017; Gérard et al., 2020). Further, results are independent of the included muscle groups. Simple pooled analysis of ECC revealed overall positive changes of +19% for eccentric, and +9% for concentric strength, +10% for FL and +9% for ROM. In contrast, CONC also shows improvements in eccentric (19%) and larger effects for concentric (16%) strength but could not improve FL (−1%) (Table 5). Therefore, there is clear evidence that ECC concurrently improves flexibility and strength parameters by combining the benefits of strengthening and stretching interventions in one exercise.

### 4.2 Interpretation of Results

The main findings of this review describe a large benefit of ECC compared to CONC. While observed strength improvements between ECC and CONC are comparable (e.g. Murphy, 2003; Duhig et al., 2019), the induced change in ROM is much larger in ECC compared to CONC which shows no changes in ROM. A comparison of studies on stretching show that the ROM improvements caused by ECC are comparable to specific stretching exercise like ballistic stretching (11%), but minor compared to static exercises (18–21%) (Thomas et al., 2018). However, since ECC shows effects for both stretching and strength, it has multiple effects and is therefore suitable for improving training efficiency. What is further unique to ECC is fascicle lengthening, which is neither induced by CONC (Gérard et al., 2020) nor by stretching (Konrad and Tilp, 2014; Lima et al., 2015).

The effects described for ECC may be explained by several mechanisms. The most important mechanism which can explain improvements of a torque-angle relationship is sarcomerogenesis (Butterfield, 2010). It leads to a longitudinal hypertrophy of muscular fascicles. This happens after a repeated overstretch of muscular structures follow by a reconstruction and addition of sarcomeres in order to avoid further traumas of the muscle within the “new” ROM (Toigo and Boutellier, 2006). Therefore, most studies examine a change in FL to describe a possible addition of sarcomeres in series. If maximum degrees of ROM get barely used, an atrophic response can follow fast (Toigo, 2019). This reaction is characterized by a significant reduction of FL after detraining (Timmins et al., 2016b). A positive change in FL is reported to be closely related to shifts in the torque-angle relationship after ECC for the vastus lateralis (Blazevich et al., 2007). This is confirmed by a second study showing that sprinting performance is positively associated with a fascicles’ length (Kumagai et al., 2000). Therefore, exercises combining stretching and strengthening, such as ECC, seem to be most effective in simultaneously increasing strength and flexibility. The result is a gain in motor performance (Kumagai et al., 2000) and a highly relevant reduction of injury risk by improving FL, strength and ROM as three of the most important risk factors (Wilk et al., 1993; Murphy, 2003; Timmins et al., 2016a). Nevertheless one study calculated the correlation between a change of FL and ROM without a significant result (Potier et al., 2009).

In contrast to the main findings and their accordance to literature, several studies showed opposite and unexpected results for ROM (Ribeiro-Alvares et al., 2018), FL (Guilhem et al., 2013; Sharifnezhad et al., 2014; Seymore et al., 2017; Kay et al., 2018), PA (Guilhem et al., 2013; Seymore et al., 2017), eccentric strength (Guilhem et al., 2013; Seymore et al., 2017), or concentric strength (Guilhem et al., 2013; Abdel-Aziem et al., 2018; Ribeiro-Alvares et al., 2018; Delvaux et al., 2020). Whereas positive ROM, FL and eccentric strength changes are common after weeks of ECC (O’Sullivan et al., 2012; Franchi et al., 2017) and a longitudinal hypertrophy seems obvious, a positive change in PA or concentric strength can be interpreted as a sign of radial hypertrophy (Butterfield, 2010; Franchi et al., 2017). Whereas most of the studies show results in accordance with literature, 2 studies (Guilhem et al., 2013; Seymore et al., 2017) do not show any relevant change in flexibility or strength and are worth discussing.

Seymore et al*.* (2017) revealed a non-significant eccentric strength change of +11.6% for ECC in contrast to −4.6% for CG. This percentage change lies in between the lowest significant result reported by Delvaux et al*.* (2020) with +7.1% and the overall average change of +19% across all included studies. To explain differences within the results, we compared the methods of training and testing which led to several possible reasons for this unusual result. At first, Seymore et al*.* (2017) examined the impact of a field exercise (NHE) on flexibility and strength resulting in lower improvements compared to exercises on a very standardized training machine such as an isokinetic dynamometer (e.g. Potier et al., 2009; Fouré et al., 2013; Franchi et al., 2014). Second, Seymore et al. (2017) show 6 weeks of intervention and within each session a load of 12 repetitions and 3 sets in total. The average training load across all included studies showed 7 weeks of training, more than 4 sets per training and more intense workouts on a weight machine (Table 3). Therefore, the chosen training load in the reported study of Seymore et al*.* (2017) is not only below average, it also appears to be below the recommended load for stimulation of longitudinal and cross-sectional hypertrophy of the skeletal muscle (Toigo and Boutellier, 2006; Butterfield, 2010; Franchi et al., 2017). Third, Seymore et al*.* (2017) only tested for isokinetic eccentric strength, which was non-significant for another 2 subgroups (Guilhem et al., 2013). In contrast to eccentric or concentric dynamic testing, isometric tests revealed significant improvements for all 15 subgroups (Table 5). It shows that an isometric test can reveal hidden information on multiple effects of ECC.

Interestingly, Guilhem et al*.* (2013) do not show any of these differences in testing and training. Nevertheless, both studies (Guilhem et al., 2013; Seymore et al., 2017) also reported no change in any flexibility parameter. A possible explanation for Guilhem et al*.* (2013) was a lack of overloaded stretch resulting in a low stimulus for sarcomergenesis. Since Sharifnezhad et al. (2014) showed that ROM and movement velocity need to be higher than usual to enhance FL, we can share the interpretation of Guilhem et al*.* (2013). At the very end of a movement when Titin has to compensate for a muscles’ continued extension, the rising stress on the muscle-tendon-unit also raises the probability for sarcomerogenesis (Butterfield, 2010). Interestingly, the unexpected and positive change of PA within both studies (Guilhem et al., 2013; Seymore et al., 2017) may have led primarily to a radial and not to a longitudinal hypertrophy (associated with a positive change in FL) of the muscle (Butterfield, 2010; Franchi et al., 2017). This may confirm the hypothesis that both training protocols included significantly fewer stimulus of flexibility training and more stimulus typical for strength training.

Another explanation for an unexpected lack of FL improvement may be the employed test method. It is a fact that ultrasound is very economic compared to other methods of measurements such as magnet resonance imaging (MRI, Oudeman et al., 2016) or even biopsy (Boakes et al., 2007). The downside is its lowered reliability (Kwah et al., 2013) compared to MRI (Oudeman et al., 2016) or an extended field of view ultrasound (EFOV, Noorkoiv et al., 2010). Especially a different handling and positioning of ultrasound can change results (Kwah et al., 2013) which may explain why Guilhem et al*.* (2013) found a +47% fascicular lenghening in pre-study observations compared to a non-significant result after ECC isokinetic training in the main study. Since a classical ultrasound may also lead to an underestimation of FL by up to +20.3% (Noorkoiv et al., 2010; Franchi et al., 2015), it can be seen as another possible reason for low changes of FL observed in both studies (Guilhem et al., 2013; Seymore et al., 2017). The fact that sarcomer length and elongation are not uniform across the muscle (Moo et al., 2016) and that a classical ultrasound allows a limited view of bigger muscles (Noorkoiv et al., 2010), emphasizes the need for MRI, EFOV, or 3D ultrasound (Uysal et al., 2021) applications.

## 5 Limitations and Recommendations

Several limits of this systematic review have been observed. Due to the reviews’ exclusive focus on healthy samples, laboratory settings and long-term interventions with RCT characteristics, some interesting trials could not be included. Especially interventional studies examining the shoulder joint (Oyama et al., 2011; Camargo, 2014; Dejaco et al., 2017; Uhl et al., 2017) did not meet the inclusion criteria. Therefore, we were not able to interpret the effects of ECC for the shoulder as planned. Exclusion of injured samples and studies with a focus on easy-to-implement interventions further limit this review. We are not able to fully understand the effectiveness of ECC implemented in frequent athletic training. In addition, we do not know the effects of ECC compared to conventional athletic training. Most studies used an isokinetic dynamometer to maximize intensity and validity of training. Based on trials of Sharifnezhad et al*.* (2014) and Marzilger et al*.* (2020) *,* it seems to be clear which ECC training stimulus is needed for improvements in FL, ROM and strength. Also, studies by Timmins et al*.* (2016b) and Blazevich et al*.* (2007) helped to unterstand the different responses after ECC or CONC and the time course of adaption.

Since Zandt et al*.* (2010) realized that there exist few ECC studies on the shoulder joint, future studies and review articles need to focus on the upper body joints. This is also relevant because of the anatomical and physiological differences between a shoulder and a knee joint which will lead to different reactions after ECC (Zandt et al., 2010). It also seems to be important to review studies comparing ECC to conventional athletic training with a focus on its consequences on sport-related performance metrics.

## 6 Conclusion

The results of 18 laboratory and multivariate studies show that ECC is a multi-effective intervention strategy for the lower limb. Not only the magnitude of change but also the amount of significant changes for several flexibility and strength metrics reveal benefits compared to classical strength training. ECC combines both stretching and strengthening in one exercise. In conclusion, especially risk factors like low eccentric strength, FL, and ROM can be improved best with ECC. It remains unclear whether this can be achieved similarly for other regions than the legs and other samples.

## Data Availability

The original contributions presented in the study are included in the article/Supplementary Material, further inquiries can be directed to the corresponding author.
